# A pilot study to examine the association between immigration experience and cognition among older Chinese Americans

**DOI:** 10.1002/alz70860_107501

**Published:** 2025-12-23

**Authors:** Li Zhou, Shreya Upendra, Mahmood Mohamed, Jessica Spat‐Lemus, Ankit Parekh, Lorene Leung, Drew Melancon, Amy Aloysi, Judith A. Neugroschl, Yiyu Cao, Linghsi Liu, Hiuyu Au, Joanne Lim, Clara Li

**Affiliations:** ^1^ Icahn School of Medicine at Mount Sinai, New York, NY, USA; ^2^ Montclair State University, Montclair, NJ, USA

## Abstract

**Background:**

Numerous studies have reported the relationships between immigration experiences and cognition in older adults who immigrated to the US across different racial/ethnic groups. However, fewer studies have examined the unique risk factors of Alzheimer's Disease and Related‐Dementias (ADRD) in older Chinese Americans who have immigrated to the US. Therefore, this pilot study aimed to investigate the association between immigration experiences and cognition among older Chinese Americans.

**Method:**

Data for this pilot study was collected at the Mount Sinai Alzheimer's Disease Research Center (ADRC). Eighty‐eight Chinese American older adults, with either normal cognition or mild cognitive impairment, were included in the analyses (mean age: 71.2±6.6 years; 29.5% Male). Information about immigration experiences ‐ including acculturation to Chinese or Western cultures, age at immigration, and duration of immigration (i.e., length of time in the U.S.), ‐ were collected via a questionnaire. Global cognition was evaluated using the Clinical Dementia Rating (CDR) scale and the Montreal Cognitive Assessment (MoCA). The sum score of CDR (CDRSOB, higher scores indicate poorer cognition) and the total score of MoCA (higher scores indicate better cognition) were used for the analyses. Linear regression models were used to examine the association between immigration experiences and cognition, while controlling for age, gender, education, and socioeconomic status.

**Result:**

Greater Chinese cultural acculturation was marginally associated with better cognitive performance, as evidenced by higher MoCA total scores (*p* = 0.056) and lower CDRSOB (*p* = 0.079). Furthermore, younger age at immigration and longer duration of immigration appeared to be associated with poorer cognition, although the result was not statistically significant.

**Conclusion:**

These preliminary findings suggest a relationship between immigration experiences and cognition among older Chinese Americans. Further research with larger samples is needed to gain a better understanding of the role of immigration in AD/ADRD risk within this historically underrepresented population.

